# Increased risk of malignant progression in benign proliferating breast lesions defined by expression of heat shock protein 27

**DOI:** 10.1038/sj.bjc.6601449

**Published:** 2004-01-06

**Authors:** P A O'Neill, A M Shaaban, C R West, A Dodson, C Jarvis, P Moore, M P A Davies, D R Sibson, C S Foster

**Affiliations:** 1 Clatterbridge Cancer Research Trust, JK Douglas Laboratories, Bebington, Wirral CH63 4JY, UK; 2 Department of Cellular and Molecular Pathology, University of Liverpool, Liverpool L69 3GA, UK; 3 Department of Public Health, University of Liverpool, Liverpool L69 3GA, UK

**Keywords:** breast cancer, neoplastic progression, heat shock protein 27

## Abstract

Heat shock protein 27 (hsp-27) is a regulator of oestrogen receptor (ER) expression and a modulator of intracellular homeostasis. In this laboratory, Shaaban *et al* demonstrated the importance of ER-*α*, together with Ki67, in enhancing the progression of benign breast lesions of defined morphological types. To better understand the mechanisms by which ER-*α* promotes breast neoplasia, this study was performed to test the hypothesis that the roles of ER-*α* and hsp-27 may be defined by their quantitative expression in proliferative breast lesions of varying histological risk. The expression of hsp-27 was identified using a specific monoclonal antibody and analysed to assess the proportion of positive epithelial cells using digitised morphometric image analysis. The expression of ER-*α* was analysed by immunohistochemistry and Western blotting in a variety of benign (HUMA121) and malignant mammary cell lines, including ER-*α*(+) (MCF7, ZR-75, T47D) and ER-*α*(−) (MDA-MB 231) breast cancer cell lines. The data confirm that, during progression from normal through proliferative breast lesions to *in situ* cancer, there was a significant increase in both the proportion and the optical density of the epithelial cells expressing hsp-27. The mean levels of expression ranged from 7.4% of the total number of epithelial cells in normal lobules to 25.17% of epithelial cells in hyperplasias of usual type (HUT) to 61.1% of epithelial cells in ductal carcinoma *in situ* (*P*<0.001). The study has confirmed the expression of hsp-27 to be closely associated with ER-*α*(+) expression, and that its regulated expression occurs early along the mammary oncogenic pathway, supporting the initial hypothesis. It is our proposal that the differential expression of hsp-27 modulates the phenotypic behaviour of morphologically benign epithelial cells and hence may be an important determinant in initiating, or promoting, a population of human mammary cancers.

There are several distinct routes by which human breast cancers of ductal type are believed to develop (Shaaban *et al*, 2002a, 2002c). One such putative pathway, from normal to invasive carcinomas, occurs through the morphological intermediaries of nonatypical hyperplasia (hyperplasia of usual type, HUT) and atypical ductal hyperplasia (ADH) to ductal carcinoma *in situ* (DCIS). The relative risk of ductal carcinoma arising in these lesions increases at each stage (Lakhani, 1999). One possible approach to understanding the mechanisms involved in mammary carcinogenesis is by comparing the expression of putative markers of risk in morphologically normal, precancerous and malignant lesions. In this respect, oestrogen-associated proteins are of particular interest since this hormone plays a crucial role in regulating mammary epithelial proliferation (Buerger *et al*, 2001), and is recognised as promoting the malignant progression of certain neoplastic and potentially premalignant lesions (Page and Dupont, 1990). Important modulators of oestrogen, and likely mediators of this hormone's effect on breast epithelial cells, act through different receptors, including the family of homeostatic regulators known as heat shock proteins (hsp's) (Kato *et al*, 2000).

A wide range of pathophysiological influences regulate the expression of hsp's in normal physiology as well as in a variety of pathological conditions. Categories of proteins within the overall family of hsp's have been classified, according to their relative molecular mobility (*M*_r_) on gel electrophoresis. In mammals, these comprise hsp's-100, -90, -70, -60 and the small molecular weight proteins (Coffer *et al*, 1985). The oestrogen-regulated protein known as hsp-27 was initially identified in breast epithelial MCF-7 cells (Edwards *et al*, 1980; Conroy and Latchman, 1996). The gene for this protein is known to produce a number of splice variants from a single gene, encoding at least 13 different protein isoforms. The expressed isoforms have multiple complementary functions, including the regulation of translational initiation and thereafter chaperone activity. Thus, the protein has an important role in maintaining intracellular protein homeostasis. The protein acts as a molecular chaperone and allows cells to survive damage from a variety of insults that may be otherwise lethal (Jakob *et al*, 1993). Thus, the modulated regulation of apoptosis might be a potentially important mechanism by which hsp-27 is permissive for, or promotes, breast carcinogenesis.

Previous work in this laboratory has confirmed hsp-27 to be an independent predictor of clinical outcome in prostate cancer (Cornford *et al*, 2000). That study found *in situ* neoplastic transformation of prostatic epithelium to be associated with transient loss of hsp-27 expression. The subsequent re-expression of hsp-27 occurred in some, but not all, invasive cancers where the quantitative level of hsp-27 expression correlated with Gleason grade, and was independently predictive of poor clinical outcomes. While the precise mechanisms remain uncertain, and are being elucidated, it is recognised that the modulated expression of ER and the initiation of apoptosis are important factors in the promotion of prostate cancer. Such a mechanism may also play a role in breast cancers, another hormone-dependent neoplasm of glandular epithelium.

While there has been considerable interest in the potential role of hsp-27 in advanced breast cancer (Thor *et al*, 1991), little is known about its expression in early breast lesions, particularly those now recognised to be associated with abnormal ER-*α* expression. A previous study by Ciocca *et al* (1983) described the expression of hsp-27 in a small number of normal breast tissues (*n*=7) and fibroadenomas (*n*=5). One further study has assessed the pattern of immunostaining of hsp-27 (also called 24-kDa protein) in a range of benign breast biopsy specimens and showed little or no staining in ducto-lobular tissue or fibroadenoma, but high levels of staining in apocrine metaplasia (Courtney *et al*, 1990). However, no previous studies have assessed the expression of hsp-27 in a large number of benign, preneoplastic and malignant breast lesions, using digitised image analysis to clarify its association with mammary neoplastic transformation. Nor has there been any previous detailed analysis of hsp-27 expression in benign or malignant mammary cell lines.

Therefore, the objective of this study was to define and compare the level of expression of hsp-27 in morphologically benign breast lesions of increased risk with that in malignant breast lesions. Since hsp-27 is an oestrogen-regulated protein, and the progression of particular abnormal breast lesions is now recognised to be oestrogen-associated (Shaaban *et al*, 2002b), a further objective was to determine whether there is a demonstrable association between hsp-27 and oestrogen receptor (ER) expression in those lesions of increased risk.

## MATERIALS AND METHODS

### Patients and specimens

All patients analysed in this study were diagnosed with benign or malignant breast disease in the Department of Cellular and Molecular Pathology at the Royal Liverpool University Hospital, during the 20-year period 1979–1999. Within the group of benign disease, 36 patient specimens contained 123 foci of HUT. An additional 31 patient specimens contained 118 foci of DCIS, with an associated invasive component. Patients diagnosed with breast cancer (*n*=69) between 1993 and 1995, who had donated tissues to the Cancer Research Tissue Bank, had 345 foci of invasive ductal carcinoma, as defined by analysis of five high-power fields using image analysis. Each focus was analysed individually. Normal breast tissues from patients undergoing reduction mammoplasty (*n*=29), continued in 73 areas of morphologically normal tissue, was submitted to detailed analysis. These data are presented in Table 2. In addition, and separately during this same time period, there were 52 cases of benign nonproliferative disease comprising apocrine metaplasia (27), blunt-duct adenosis (19), sclerosing adenosis (7), fibroadenomas (5) and papillomas (3). The details of these cases are included in Table 3.

The histological preparations from all cases were reviewed independently by three investigators (PAO'N, AMS and CSF) before a consensus agreement was obtained to confirm the diagnosis and to clarify the benign and malignant lesions, according to the pathology guidelines of the UK NHS Breast Screening Programme (Sannino and Shousha, 1994).

### Tissue immunohistochemistry

#### Antibodies

Human hsp-27 was identified using a specific anti-hsp-27 mouse monoclonal antibody (Novocastra, Newcastle-upon-Tyne, UK) at a dilution of 1 : 20. ER-*α* was identified using a mouse monoclonal anti-ER-*α* antibody (clone 1D5, Dako Ltd., Ely, Cambridge, UK) at a dilution of 1 : 30. Both antibodies were diluted in Tris-buffered saline (TBS) comprising 50 mM Tris (pH 7.4) containing 8% (w v^−1^) NaCl and 5% (w v^−1^) bovine serum albumin.

### Immunostaining methods

Immunostaining was performed using the standard technique previously described (Cornford *et al*, 2000). Paraffin wax-embedded tissue sections were dewaxed with xylene, rehydrated through graded ethanols followed by blocking of endogenous peroxidase activity in H_2_O_2_/methanol for 12 min. For hsp-27 and for ER-*α* immunohistochemistry, antibody-binding epitopes were retrieved by pressure-cooking tissue sections for 2.5 min and then, after cooling to room temperature, incubating sections with the appropriate antibody for 40 min. After washing twice with TBS, slides were incubated with sheep anti-mouse immunoglobulin (Dako, Envision) for 30 min. Sections were then washed twice before immersion in a solution of DAB (2 mg ml^−1^) in phosphate-buffered saline (PBS) (pH 7.4) for 10 min. All incubations were performed at room temperature. Washes with TBS were performed between each step. Nuclei were counterstained with Meyer's haemalum before mounting slides in DPX.

### Controls

Negative immunohistochemical controls were included by performing identical immunohistochemical procedures, but substituting irrelevant nonimmune serum for the primary antibody. Three different prostatic carcinomas of varying intensities of hsp-27 expression were used as positive controls in each batch of staining. Three ER-*α*(+) breast carcinomas were similarly used as positive controls.

### Assessment of immunostaining

#### Heat shock protein 27

For each case of invasive cancer, the number of positively stained epithelial cells was estimated visually by scanning the entire tissue at low power using conventional light microscopy. At least five high-power fields per case were analysed and expressed as a percentage of the total epithelium present. The mean percentage was calculated for every case. Initially, for control normal tissue sections, together with any benign changes or DCIS, the percentage of positively stained cells was counted for each type of focus and then averaged to give a mean percentage per case.

All tissue sections assessed visually were submitted to digitised image analysis to measure the mean optical density (OD) of staining. Prior to each set of measurements, and at intervals during each session, the optical system was calibrated using standard slides. On each occasion, the microscope was calibrated using identical parameters and the OD of the standard slide was recorded. For all control normal tissues, for benign lesions and for DCIS, each focus was individually assessed and the OD averaged for each case. For invasive cancers, the OD of immunostained malignant cells was assessed on five high-power fields and the mean number of positive cells calculated for each case.

#### Oestrogen receptor-*α*

The percentage of stained epithelial cells was calculated as a proportion of the total number of epithelial cells present on the slide. A 10% cut-off was applied as the criterion to define positive staining for invasive cancers (Sannino and Shousha, 1994), also conforming to our previous studies (Shaaban *et al*, 2002a).

### Epithelial cell lines

Cell lines employed in this study, and their properties, are summarised in Table 1

**Table 1:** Characteristics of mammary cell lines included in the study

**Cell line**	**Origin**	**Morphology**	**ER-*α* status**	**Reference**
HUMA121	Benign breast disease^a^	Epithelial	Negative	Rudland *et al* (1993)
MCF7	Breast carcinoma	Epithelial	Positive	Soule *et al* (1973)
ZR-75	Breast carcinoma	Epithelial	Positive	Engel *et al* (1978)
T47D	Breast carcinoma	Epithelial	Positive	Westley and Rochefort (1980)
MDA-MB 231	Breast carcinoma	Myoepithelial-like	Negative	Cailleau *et al* (1974)

a Parent cell line: HMT3522 (Rudland *et al* 1993).

. HUMA121 was supplied by Professor P Rudland, University of Liverpool; MCF7 and ZR-75 were supplied by Mr M Shaw, ICI Pharmaceuticals Ltd., Macclesfield; T47D was supplied by Professor I Keydar, Faculty of Life Sciences, University of Tel Aviv; MDA-MB 231 was a gift from Dr C Green, University of Liverpool.

### Cell culture

Cells were cultured as monolayers in DMEM (Life Technologies, Inc.) supplemented with 10% (v v^−1^) foetal calf serum (Life Technologies, Inc., Paisley, Scotland), 1 mM glutamine, 100 IU ml^−1^ penicillin G and 100 IU ml^−1^ streptomycin in an atmosphere of 5% CO_2_ in air at 100% humidity and 37°C. The T47D cell line was supplemented with insulin at a concentration of 1 *μ*g ml^−1^. Media were changed on alternate days.

### Immunohistochemistry of cell lines

The expression of hsp-27 by human benign mammary cell line HUMA121 (Rudland, 1987), ER-*α*(+) breast cancer cell lines (MCF7, ZR-75, T47D) and ER-*α*(−) breast cancer cell line (MDA-MB 231) was analysed by immunohistochemistry: Monolayered cultures and cell blocks were prepared from the five cell lines. Monolayered cultures were obtained by allowing cells to grow on sterile glass slides until subconfluent and then fixed by immersion in 95% (v v^−1^) methanol for 5 min. Paraffin-wax-embedded cell blocks were prepared from 2% (w v^−1^) agar using the following protocol: After trypsinisation, cells were washed with two changes of PBS (20 mM, pH 7.2), followed by scraping using a rubber policeman followed by centrifugation at 2500 rpm for 3 min. Cells were then fixed overnight using 10% (v v^−1^) buffered formal saline, followed by centrifugation at 2500 rpm for 10 min. After discarding the supernatant, molten agar was added to the cell pellet, which was centrifuged and allowed to solidify at 4°C. The pellet was processed routinely and embedded in paraffin-wax blocks. Sections were cut at 5 *μ*M and stained immunohistochemically for hsp-27. Smears were stained manually using the identical hsp-27 antibody, but without antigen retrieval. Washings were performed between all stages of the procedure using TBS-Tween.

### Gel electrophoresis and Western blotting

The expression of hsp-27 by human benign mammary cell line HUMA121 (Rudland, 1987), ER-*α*(+) breast cancer cell lines (MCF7, ZR-75, T47D) and ER-*α*(−) breast cancer cell line (MDA-MB 231) was analysed by polyacrylamide gel electrophoresis and Western blotting: Cell lysates were prepared in a buffer comprising 10 mM Tris (pH 6.8) containing 0.5% sodium deoxycholate, 0.02% sodium azide, 20% (v v^−1^) glycerol, 0.5% Triton X-100 and 100 *μ*g ml^−1^ phenylmethylsulphonyl fluoride (PMSF). Initially, 0.5 ml of lysis buffer was spread over each 9-cm culture plate of cells, incubated for 15 min to obtain cell lysis before the cells were scraped to one edge of the plate and aspirated into a microfuge tube. The lysate was centrifuged at 3000 rpm for 10 min. The supernatant was aspirated and transferred to a fresh microfuge tube. The protein concentration of the supernatant was measured using the Bradford assay (Bio-Rad Laboratories Ltd., Hemel Hemstead, UK). Equal amounts of protein (20 *μ*g per lane) were separated by 12% (w v^−1^) SDS/PAGE. To verify that equal amounts of proteins were loaded in the stacking gels, the actin expression was estimated in each lane using a rabbit polyclonal anti-actin antibody (Santa Cruz Biotechnology, Inc., UK). After separation, proteins were transferred onto nitrocellulose membranes by electroblotting. Thereafter, blots were blocked by immersion at room temperature for 1 h in ProtoBlock solution (National Diagnostics Inc., UK) and probed with the identical antibody used for immunohistochemistry and at the same dilution. After three 15-min washes in 10 mM Tris-buffer (pH 7.5), blots were incubated with peroxidase-conjugated secondary antibodies. The enhanced chemiluminesence Western blotting analysis system (Amersham Life Science) was used for protein detection. Blots were repeated twice to confirm the data.

### Statistical methods

All data were analysed by Spearman's rank correlation coefficient (*r*) using SPSS software for Windows (release 10.0). Fisher's *z*-transformation test was used to compare any pair of correlation coefficients. Student's *t*-test was used to compare the means of the percentage expression and optical density among the different groups. *P*⩽0.05 was used to define statistical significance.

## RESULTS

### Heat shock protein *27*

#### Control normal epithelium

All control normal specimens expressed hsp-27 by some cells in each case. However, the expression of hsp-27 was heterogeneous within these tissues. A wide variation was found in the percentage and intensity of epithelial cell staining within adjacent foci (Table 2

**Table 2:** hsp-27 expression (mean % and mean OD) in normal, benign and malignant breast

**Lesion**	**Cases (*n*)**	**Foci (*n*)**	**Mean % positive cells (±s.d.)**	**Mean O.D.±s.d.**
Normal	29	73	7.40±11.8	0.34±0.22
HUT	36	123	25.17±24.68^a^	0.50±0.23^b^
DCIS	31	118	61.10±31.47^c^	0.63±0.18^d^
IC	69	345	58.87±36.5^e^	0.56±0.12^f^

a Higher than normal *P*<0.001.

b Higher than normal *P*=0.008.

c Higher than HUT *P*=0.008 and normal *P*<0.001.

d Higher than HUT *P*=0.01 and normal *P*<0.001.

e Lower than DCIS *P*=0.62, higher than HUT (*P*<0.001) and higher than normal (*P*<0.001).

f Lower than DCIS *P*=0.22 , higher than HUT(*P*=0.596)and higher than normal (*P*<0.001).

**Figure 1:**
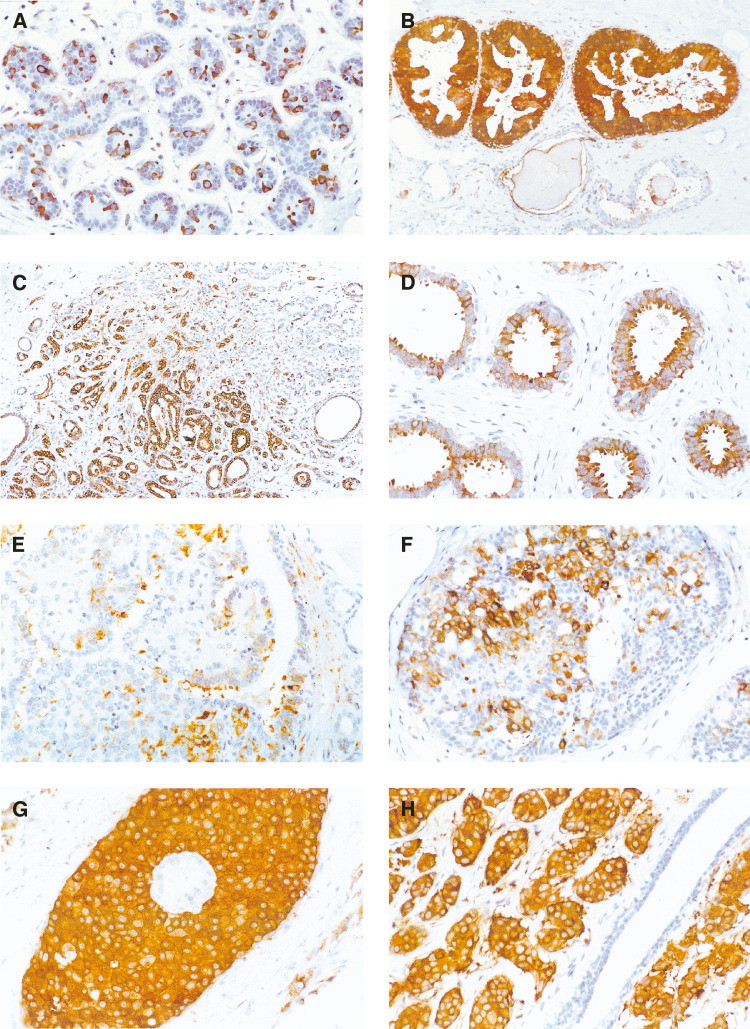
Patterns of hsp-27 expression in different breast lesions: (**A**) hsp-27 expression in normal terminal duct lobular unit showing scattered positive cells. Note that the expression is cytoplasmic with no nuclear staining. (**B**) Consistently high level of expression in apocrine metaplasia with negative staining of adjacent normal ducts. (**C**) BDA showing cytoplasmic staining with characteristic apical localisation within cytoplasmic snouts. (**D**) hsp-27 expression in sclerosing adenosis showing heterogeneity in % and intensity of staining. (**E**) Staining in papilloma is localised to the epithelium with no expression in the stroma. (**F**) hsp-27 expression in HUT consisting of more frequent positively stained epithelial cells than in normal lobules. (**G**) DCIS with a large number of strongly stained cells. (**H**) Uniformly strong positive staining in invasive cancer with adjacent nonstained normal duct.

HUT=hyperplasia of usual type. DCIS=ductal carcinoma *in situ*. IC=invasive carcinoma I.

). The distribution of hsp-27 was predominantly cytoplasmic and diffuse, although occasionally there was granularity of the cytoplasm and/or staining of plasma membranes (Figure 1A

). The mean proportion of epithelial cells expressing hsp-27 was 7.4% with a mean OD of 0.32. A strong positive correlation was found between the mean percentage of epithelial cells expressing hsp-27 and the corresponding OD of its expression (*r*=0.505, *P*=0.005).

#### Benign epithelium – nonproliferative lesions

In all these lesions, expression occurred within the epithelium, with no staining of the stroma. These data are summarised in Table 2. In all cases of epithelial apocrine metaplasia, hsp-27 was consistently expressed within the cytoplasm (Figure 1B). In Blunt duct adenosis (BDA) (Table 3

**Table 3:** hsp-27 expression in nonmalignant breast lesions

**Lesion**	**Positive cases (*n*)**	**No. of foci (*n*)**	**Positive cases (%)**	**Mean % hsp-27 positive cells**
Apocrine metaplasia	27	27	100	100
BDA^a^	16	19	84.21	47.5
Sclerosing adenosis	4	7	57.14	53
Fibroadenoma	3	5	60	75
Papilloma	2	3	66.67	22

a BDA=blunt duct adenosis.

), hsp-27 was expressed in most foci where it occurred predominantly within the cytoplasm of epithelial cells, characteristically localised to apical cytoplasmic ‘snouts’ (Figure 1C). Sclerosing adenosis (Figure 1D), papillomas (Figure 1E) and fibroadenomas variably expressed hsp-27 within the contained epithelial component.

#### Benign epithelium – hyperplasia of usual type

The mean percentage of hsp-27 expression (25.17±24.68) was higher in HUT (Figure 1F) when compared with normal foci (*P*<0.001), as was the mean OD of staining (*P*=0.008).

#### Malignant epithelium

**Figure 2:**
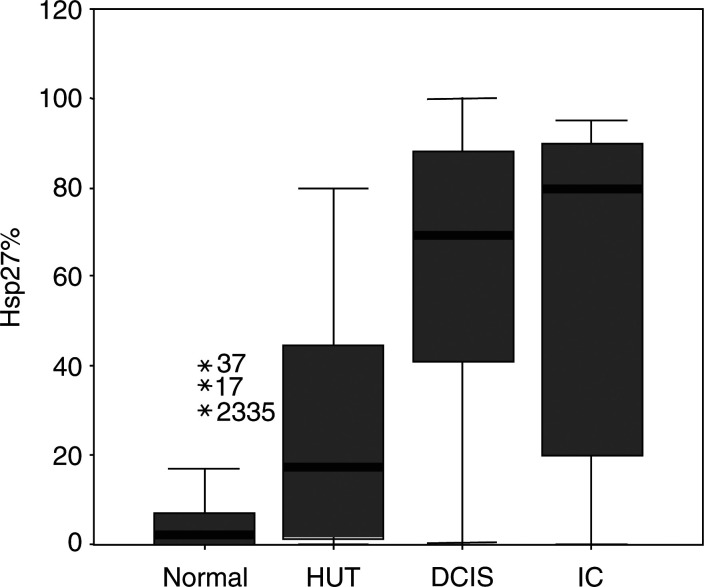
Boxplot graph plotted for the percentage expression of hsp-27 in the normal breast, HUT, DCIS and invasive cancer.

**Figure 3:**
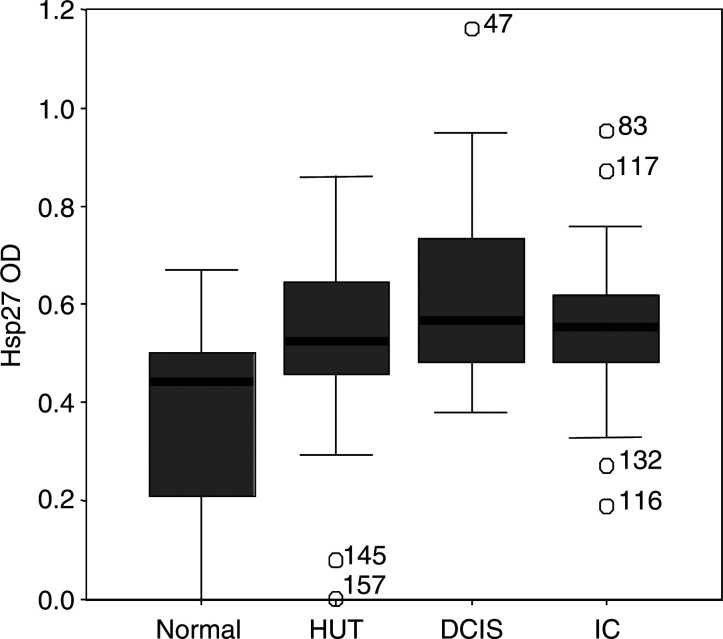
Boxplot graph plotted for the optical density of hsp-27 immunostaining in the normal breast, HUT, DCIS and invasive cancer.

The mean percentage of hsp-27 expression was higher in DCIS (Figure 1G) when compared with HUT (*P*<0.001; Figure 2

). Similarly, the mean optical density (OD) of hsp-27 was significantly increased from HUT to DCIS (*P*=0.01; Figure 3

). However, no significant difference was identified in either the proportion of cells expressing hsp-27 or the OD of staining when comparing low-grade DCIS (*n*=16) with high-grade DCIS (*n*=15). Also, there was no significant difference in the mean percentage of hsp-27 expression by epithelial cells or mean OD of staining between DCIS and invasive cancer (Table 2).

With respect to intercellular distribution, heterogeneity was a feature of staining commonly observed within invasive cancers (Figure 1H), with some cases containing intensely stained regions immediately adjacent to negative regions. A strong positive correlation was found between the mean proportion of cells expressing hsp-27 and the OD of staining in normal lobules (*r*=0.505, *P*=0.005), HUT (*r*=0.679, *P*<0.001) and invasive cancers (*r*=0.646, *P*<0.001). However, none of these values for ‘*r*’ between the groups was significantly different from one another.

### Oestrogen receptor-alpha (ER-*α*)

#### Control normal epithelium

In normal lobules, ER-*α*(+) cells were less interspersed among a majority of nonstained cells. The mean percentage of ER-*α* expression in normal breast lobules was 22.84 (±16.69). No correlation was found between ER-*α* expression and either percentage of epithelial cells expressing hsp-27 or the OD of hsp-27 staining (Table 4

**Table 4:** Relationship between ER-*α* and hsp-27 in different breast lesions

**Lesion**	**ER-*α*%(±s.d.)**	***r*_s_ (ER-*α*% and hsp%)**	***P*-value**	***r*_s_ (ER-*α*% and hsp OD)**	***P*-value**
Normal	22.84 (17.69)	−0.119	0.573	0.067	0.751
HUT	41.62 (24.19)	0.450	0.016*	0.375	0.035*
DCIS	58 (41.64)	0.824	<0.001**	0.871	<0.001**
IC^a^	57.52 (38.89)	0.450	<0.001**	0.254	0.008**

* Significant at *P*⩽0.05.

** Significant at *P*⩽0.01.

a IC=Invasive carcinoma.

).

#### Benign epithelium – nonproliferative lesions

The expression of ER-*α* was not analysed in this group since their risk of progressing to breast cancer is low, and has not been quantified. Furthermore, some of these lesions (e.g. apocrine metaplasia) are known to be ER-*α*(−).

#### Benign epithelium – hyperplasia of usual type

In HUT, the mean percentage of ER-*α* was 41.62 (±24.19; Table 4). A significant positive correlation was found between the proportion of cells expressing ER-*α* and with both the proportion of cells expressing hsp-27 (*P*=0.016) and the OD of hsp-27 staining (*P*=0.035).

#### Malignant epithelium

The mean percentage of ER-*α* expression in DCIS and invasive carcinoma was 58.01 (±41.64) and 57.52 (±38.89), respectively (Table 4). In both types of lesions, there was a highly significant correlation between hsp-27 expression and OD as well as between hsp-27 expression and ER-*α* status (*P*<0.0001) and the proportion of ER-*α*(+) cells (Table 4). The mean OD of hsp-27 staining (0.611) was significantly higher in ER-*α*(+) tumours when compared with ER-*α*(−) tumours (0.514, *P*<0.0001). Comparison between the values of ‘*r*’ in normal lobules and HUT, using Fisher's *z*-transformation, revealed a difference that almost reached significance (*P*=0.07). A similar comparison between normal lobules and invasive cancers confirmed a difference that was statistically significant (*P*=0.04). However, no statistically significant difference was found on comparing values of ‘*r*’ for HUT and invasive cancer (*P*=0.9).

#### Immunohistochemistry of cell lines

When compared with benign cell line HUMA121, a strikingly higher expression of hsp-27 occurred in the four malignant cell lines MCF7, ZR-75, T47D and MDA-MB 231. Moreover, the expression of hsp-27 was strong in ER-*α*(+) cell lines MCF7, ZR-75 and T47D, whereas there was minimal expression by ER-*α*(−) cell line MDA-MB 231.

#### Western blotting of cell lines

**Figure 4:**
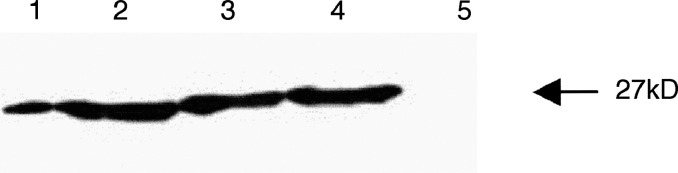
Quantitative Western blot analysis of hsp27: lane 1, MDA-MB 231; lane 2, MCF7; lane 3, ZR-75; lane 4, T47D and lane 5, HUMA121. The highest protein content occurred in ER-*α*(+) cell lines. Molecular weight marker: Triose phosphate isomerase: *M*_r_ 26 600 kDa.

Mammary cell lines exhibited marked differences in the hsp-27 expression between the benign and malignant lines. The benign cell line, HUMA121, did not express the protein. However, all four breast cancer cell lines exhibited enhanced expression, particularly ZR-75 and T47D, both of which are strongly ER-*α*(+) (Figure 4

).

## DISCUSSION

This study has extended our previous analysis of the relationship between ER-*α* and ER-associated proteins, specifically hsp-27 in human breast tissues containing morphological lesions of recognised malignant potential as well as in human breast carcinomas. In this current study, the mean expression of hsp-27 increased progressively from normal through proliferative breast disease to *in situ* cancer. However, there was no significant additional increase in the expression of hsp-27 from *in situ* cancer to invasive malignancy. Our current findings support the hypothesis that the modulated expression of hsp-27 occurs relatively early along the oncogenic pathway in mammary epithelial cells. Such alterations in expression might be an important factor contributing to the initiation of tumorigenesis. Since there is evidence that the expression of hsp-27 blocks apoptosis induced by a wide range of stimuli (Richards *et al*, 1996) and protects tumour cells against the apoptotic effects of TNF-*α* (Wang *et al*, 1996), a likely mechanism is by imparting resistance to apoptosis and then by maintaining those particular populations of epithelial cells of enhanced malignant potential.

This study has also detailed the pattern of hsp-27 expression in a range of benign breast lesions, including those suggested by some authors to carry an increased risk of developing breast cancer (Dupont and Page, 1985). In apocrine metaplasia (Courtney *et al*, 1991), the previously reported high expression of hsp-27 has been confirmed. However, the precise significance of this phenomenon is still not clear since it is believed that these lesions are ER-(−), while positive for androgen receptors (Gatalica, 1997). Furthermore, the likelihood of these lesions progressing to malignancy remains in dispute.

Although the level of expression of hsp-27 now detected in the various benign lesions is higher than previously reported (Ciocca *et al*, 1983), the antibodies employed in these earlier studies are not identical. Furthermore, current immunohistochemical techniques are more sensitive and more objective than those previously employed. Using these novel reagents, this current study has demonstrated for the first time a correlated increase in hsp-27 OD of staining from normal epithelium through nonatypical epithelial proliferations to overt malignancy. Thus, the level of expression of hsp-27 protein, in conjunction with the number of positively stained cells, might be an important factor relative to the level of ER-*α* in determining the outcome of individual benign lesions.

Hsp27 is synthesised under oestrogen control (Edwards *et al*, 1981a). The protein was originally identified in MCF-7 cells (Edwards *et al*, 1981a, 1981b) as well as in primary human breast carcinomas (Tandon *et al*, 1990). In cultured human breast cancer cells, the hsp-27 gene is regulated by oestradiol as well as by heat shock (Fuqua *et al*, 1989). Oestrogens increase the transcription of srp-mRNA by two- to three-fold, whereas antioestrogens decrease expression in an ER-positive cell line (Thor *et al*, 1991). Thus, Hsp-27 may be a component of the ER machinery (Coffer *et al*, 1985) possibly, as part of a direct feedback loop. This study has confirmed a close association between hsp-27 and ER-*α* in both proliferative epithelium and in established breast cancers. The finding that hsp-27 expression in the benign mammary cell line was barely detectable, while remarkably high in malignant cell lines, strongly supports the data from the intact tissues in which a progressive increase in hsp-27 expression was identified to extend from normal through benign to malignant specimens. In addition, the association between the expression of hsp-27 protein and ER-*α* protein, both at the cellular and tissue levels, supports the hypothesis that both proteins interact to modulate cellular functions. However, this association was not maintained in normal lobules. These findings support the concept that hsp-27 is intimately linked to oestrogen action (Adams and McGuire, 1985; Thor *et al*, 1991; Kato *et al*, 2000), although the exact mechanisms of this link remain poorly understood. The observation that ER-*α*(+) tumours expressed higher levels of hsp-27, as measured by OD when compared with ER-*α*(−) cancers, might further suggest a prognostic role for overexpressed hsp-27 in breast cancer. Quantitative digital analysis of the intensity of hsp-27 expression, as measured by OD, objectively enhances the assessment of the percentage expression by conventional visual methods. Although measurement of the OD has provided objective quantitative data on the amount of hsp-27 expressed, which is recognised to be difficult to assess using light microscopy, its precise significance remains to be established.

Increased hsp-27 expression in proliferative epithelial lesions with increased potential of malignant progression, as measured by percentage expression and OD, as well as its strong association with ER, might highlight a hormone-dependent pathway of human mammary carcinogenesis through oestrogen-dependent overexpression of hsp-27, which protects tumour cells against apoptosis. Moreover, hsp-27 may also function as a molecular chaperone in the signal transduction pathways of different cell regulators (Ciocca *et al*, 1993), thus enhancing the proposed mechanism of protection. Further large-scale studies on cohorts of benign and malignant breast lesions together with longterm follow-up and detailed molecular analysis will assist in clarifying the prognostic role and proposed mechanism(s) of action of hsp-27, particularly as a regulator of ER function, in these preneoplastic lesions.
